# Cellular Senescence in Ocular Surface Diseases: Molecular Mechanisms and Therapeutic Perspectives

**DOI:** 10.3390/ijms27209003

**Published:** 2026-10-11

**Authors:** Linkang Zhang, Shipeng Li, Qiao Gao, Hongkun Zhang, Hong Zhang, Shudan Wang

**Affiliations:** 1 Eye Hospital, The First Affiliated Hospital of Harbin Medical University, Harbin 150001, China; 2 Department of Ophthalmology, The Second Affiliated Hospital of Harbin Medical University, Harbin 150001, China; 3 Second Affiliated Hospital of Heilongjiang TCM University, Harbin 150001, China

**Keywords:** cellular senescence, senescence-associated secretory phenotype, stress-induced premature senescence, ocular surface disease, senolytics, senomorphics

## Abstract

Cellular senescence is characterized by stable cell-cycle arrest and a senescence-associated secretory phenotype (SASP). Recurrent desiccating, oxidative, ultraviolet, microbial, and immune-mediated insults may induce stress-induced premature senescence (SIPS) in ocular surface tissues. PubMed and Web of Science were searched from database inception to 30 June 2026. Primary human, animal, and in vitro studies were prioritized, and direct evidence of senescence was distinguished from indirect stress-related or SASP-like associations. The p53–p21^CIP1^–pRB and p16^INK4a^–pRB pathways mediate senescence-associated cell-cycle arrest. Reactive oxygen species (ROS)–p38 mitogen-activated protein kinase (p38 MAPK) and cyclic GMP–AMP synthase–stimulator of interferon genes (cGAS–STING) signaling act as stress-sensing and inflammatory pathways, whereas mechanistic target of rapamycin (mTOR) and AMP-activated protein kinase (AMPK) contribute to SASP and metabolic regulation. Transient senescence may support wound repair and immune clearance, whereas persistent senescence may contribute to epithelial and endothelial dysfunction, tear-film instability, immune dysregulation, fibrosis, and extracellular matrix remodeling. Mechanistic support is strongest in selected experimental models; evidence in several human ocular surface disorders remains associative. Cellular senescence is a context-dependent contributor to ocular surface pathology. Senolytic and senomorphic strategies remain predominantly preclinical and require disease-, cell-, and stage-specific validation.

## 1. Introduction

The ocular surface is an integrated functional unit comprising the tear film, cornea, conjunctiva, limbus, lacrimal and meibomian glands, eyelids, and associated neural and immune components [1,2]. Its direct interface with the external environment exposes it to recurrent desiccating, oxidative, ultraviolet, microbial, mechanical, and immune-mediated stressors [3]. Disruption of this homeostasis contributes to ocular surface diseases (OSDs), including dry eye disease, meibomian gland dysfunction, blepharitis, keratoconjunctival disorders, ocular graft-versus-host disease, conjunctivochalasis, and pterygium [3,4]. Because senescence-associated changes may affect different corneal compartments, this review also includes keratoconus, Fuchs endothelial corneal dystrophy, and corneal allograft rejection as selected conditions with direct, supportive, or indirect evidence [5]. This broader cornea–ocular surface framework permits comparison across epithelial, stromal, endothelial, glandular, and immune compartments.

Cellular senescence is generally defined as a stable state of proliferative arrest accompanied by distinct molecular and secretory changes [6,7]. Under certain conditions, external stimuli such as oxidative stress, deoxyribonucleic acid (DNA) damage, and radiation can prematurely activate intracellular senescence cascades, forcing cells to cease division and enter a senescent state; this process is referred to as stress-induced premature senescence (SIPS) [8,9].

Senescent cells develop a senescence-associated secretory phenotype (SASP), characterized by secretion of cytokines, chemokines, growth factors, and extracellular matrix (ECM)-degrading proteases. The SASP can maintain the senescent state and induce senescence in neighboring healthy cells through paracrine signaling [10,11,12]. Although ocular surface tissues possess features of immune privilege, chronic exposure to environmental and immune stressors can trigger SIPS, allowing senescent epithelial, stromal, endothelial, glandular, and immune cells to propagate bystander senescence and reshape the local microenvironment [13]. Previous reviews examined cellular senescence in ocular aging or across a broad range of ocular diseases [13,14]. This review focuses on SIPS in corneal and ocular surface homeostasis and disease. It integrates cell-type-specific mechanisms, SASP-mediated immune and stromal remodeling, and disease-specific evidence grading to assess the rationale and limitations of senescence-targeted therapy.

## 2. Literature Search and Evidence Evaluation

This narrative review was informed by a structured search of PubMed and Web of Science from database inception to 30 June 2026. Search terms combined “cellular senescence”, “stress-induced premature senescence”, “senescence-associated secretory phenotype” or “SASP” with terms related to the cornea and ocular surface, including “cornea”, “ocular surface”, “dry eye”, “Fuchs endothelial corneal dystrophy”, “ocular graft-versus-host disease”, “corneal transplantation”, “keratoconus”, “pterygium”, and “conjunctivochalasis”.

Titles and abstracts were screened for relevance, potentially eligible articles were assessed in full text, and reference lists of relevant articles were checked for additional studies. Disease-specific evidence evaluation focused on primary human, animal, and in vitro studies examining cellular senescence or potentially related stress responses in corneal or ocular surface disorders. Selected reviews and meta-analyses were included only to provide epidemiological or conceptual context and were not considered direct evidence of cellular senescence. Non-ocular studies were cited selectively to discuss general senescence mechanisms or candidate therapeutic pathways but were excluded from disease-specific evidence grading.

Evidence was qualitatively categorized as direct, supportive, or indirect. Direct evidence was based on complementary features of cellular senescence, particularly stable cell-cycle arrest or reduced proliferative capacity together with established senescence markers and, when available, SASP characterization or functional manipulation. Supportive evidence included senescence-associated markers or pathways without full phenotypic confirmation, whereas indirect evidence comprised oxidative stress, inflammatory or SASP-like changes, or tissue remodeling without direct demonstration of senescent-cell accumulation or stable cell-cycle arrest.

## 3. Mechanisms of Cellular Senescence

### 3.1. Senescence-Inducing Stressors

Cellular senescence can be induced by a variety of intrinsic and extrinsic stressors, including oxidative stress, DNA damage, telomere dysfunction, mitochondrial dysfunction, ultraviolet radiation, desiccation, and persistent inflammatory or immune-mediated injury. These stressors are particularly relevant to stress-induced premature senescence (SIPS), in which sustained cellular damage activates stress-response pathways that ultimately promote stable cell-cycle arrest and broader senescence-associated phenotypic changes. Among these upstream responses, reactive oxygen species (ROS), the DNA damage response (DDR), and stress-responsive kinases such as p38 mitogen-activated protein kinase (p38 MAPK) serve as important links between cellular injury and downstream senescence programs [8,15,16,17,18,19].

Oxidative stress is particularly relevant to ocular tissues because of their continuous exposure to environmental stress and their high metabolic demand. Excessive ROS can induce DNA damage, impair mitochondrial function, destabilize telomeres, and activate p38 MAPK and p53-related stress responses [9,16,17,19]. Persistent DNA damage activates the DDR, which can promote cell-cycle arrest while also engaging inflammatory signaling pathways associated with senescence [18,19]. In dry eye disease (DED), desiccating and oxidative stress are associated with epithelial dysfunction, inflammatory signaling, and p38 MAPK-dependent activation of matrix metalloproteinase-9 (MMP-9) [20,21]. Although increased p38 MAPK activity and MMP-9 expression alone do not establish cellular senescence, they provide a mechanistic link between chronic ocular-surface stress and pathways capable of promoting a senescence-associated state. In conjunctivochalasis (CCH), more direct evidence supports oxidative stress-induced senescence of conjunctival fibroblasts through the ROS–p38 MAPK–p53–p21^CIP1^–pRB pathway [22]. Ultraviolet exposure and chronic oxidative stress are also prominent features of pterygium, whereas persistent inflammation and immune-mediated tissue injury may contribute to senescence-associated changes in ocular graft-versus-host disease (oGVHD). In Fuchs endothelial corneal dystrophy (FECD), oxidative stress, mitochondrial dysfunction, and DNA damage have likewise been implicated in corneal endothelial cell dysfunction and senescence-related phenotypes [22,23,24,25,26].

Persistent DNA damage and mitochondrial dysfunction may additionally promote the accumulation of cytosolic DNA. Nuclear DNA released from damaged nuclei or micronuclei, as well as mitochondrial DNA released from dysfunctional mitochondria, can activate cyclic GMP–AMP synthase–stimulator of interferon genes (cGAS–STING) signaling. Activation of this pathway promotes type I interferon and NF-κB-associated inflammatory responses and therefore provides an additional mechanistic connection between intracellular damage sensing, inflammation, and cellular senescence [17,27,28]. However, current evidence for cGAS–STING-mediated senescence is derived predominantly from general or non-ocular models, and its direct contribution to ocular-surface senescence remains to be established.

### 3.2. Cell-Cycle Arrest Effector Axes

Stable cell-cycle arrest is a defining feature of cellular senescence and is mediated primarily by the p53–p21^CIP1^–pRB and p16^INK4a^–pRB pathways [6,29]. DNA damage, oxidative stress, telomere dysfunction, and replication stress can activate the DNA damage response (DDR) and downstream p53 signaling, thereby increasing p21^CIP1^ expression [9,19,30,31,32,33]. p21^CIP1^ inhibits cyclin-dependent kinase (CDK) activity, reduces pRB phosphorylation, and maintains pRB in a hypophosphorylated state that suppresses E2F-dependent transcription and blocks G1/S progression. Persistent oxidative or genotoxic stress can further reinforce this response through p38 MAPK signaling.

The p16^INK4a^–pRB pathway is another major effector of senescence-associated cell-cycle arrest. p16^INK4a^ inhibits CDK4/6, maintains pRB in its growth-suppressive hypophosphorylated form, and prevents E2F-mediated entry into S phase. Although the p53–p21^CIP1^–pRB and p16^INK4a^–pRB pathways converge on pRB-dependent cell-cycle control, their relative contributions and temporal dynamics vary by cell type, senescence inducer, and disease context [34,35].

In ocular tissues, these arrest pathways link chronic stress to functional decline [13,14]. Increased *CDKN1A* expression has been reported in Fuchs endothelial corneal dystrophy (FECD)-related corneal endothelial models, increased p16^INK4a^ protein expression occurs in chronic ocular graft-versus-host disease (oGVHD) tissues, and p53–p21^CIP1^–pRB signaling has been implicated in oxidative stress-induced senescence of conjunctivochalasis (CCH) fibroblasts [22,23,24,25,26]. These findings indicate that the dominant effector pathway may differ across ocular cell types and disease conditions.

### 3.3. SASP Regulation and Immune Interactions

In addition to stable cell-cycle arrest, senescent cells undergo extensive changes in secretion. The SASP is regulated by interconnected signaling networks, among which p38 MAPK and nuclear factor kappa B (NF-κB) are major transcriptional regulators. Persistent stress and DNA damage can activate NF-κB-dependent programs and promote expression of SASP-associated factors [8,15,16,18]. Increased inflammatory or SASP-associated signaling alone, however, is insufficient to establish cellular senescence without stable cell-cycle arrest and complementary senescence markers.

The SASP is highly heterogeneous and varies according to cell type, senescence inducer, tissue microenvironment, and duration of senescence. Its components include pro-inflammatory cytokines such as interleukin-1 alpha (IL-1α), interleukin-1 beta (IL-1β), IL-6, IL-8, and tumor necrosis factor-alpha (TNF-α); chemokines such as C-C motif chemokine ligand 2 (CCL2), C-C motif chemokine ligand 5 (CCL5), and C-X-C motif chemokine ligand 8 (CXCL8); growth factors such as transforming growth factor-β (TGF-β), vascular endothelial growth factor (VEGF), and granulocyte-macrophage colony-stimulating factor (GM-CSF); and matrix-remodeling enzymes such as MMP-1, MMP-3, and MMP-9 [12,36]. Through autocrine signaling, these factors can reinforce the senescent phenotype of the producing cell, whereas paracrine signaling can alter the behavior of neighboring epithelial cells, fibroblasts, endothelial cells, and immune cells.

SASP-associated signaling can also propagate senescence to neighboring cells through IL-1α/NF-κB, signal transducer and activator of transcription 3 (STAT3), and TGF-β-related pathways, thereby contributing to the spatial expansion of senescence within tissues [10]. At the same time, SASP-derived chemokines can recruit monocytes/macrophages, neutrophils, and T cells and thereby reshape the local immune microenvironment. This immune recruitment may initially promote the recognition and clearance of senescent cells, whereas persistent SASP signaling can sustain chronic inflammation, promote secondary senescence, and contribute to a self-perpetuating cycle of immune activation and tissue injury [37].

Senescent cells may also acquire immune-evasive features that limit their clearance. One proposed mechanism involves upregulation of programmed death-ligand 1 (PD-L1), which can interact with programmed cell death protein 1 (PD-1) on immune cells and attenuate immune effector responses [38,39,40]. More broadly, impaired immune surveillance and immune-evasion mechanisms may facilitate the persistence and accumulation of senescent cells within tissues, thereby prolonging SASP-associated inflammatory and tissue-remodeling effects [37,41,42,43]. However, the extent to which these mechanisms operate in ocular-surface tissues remains incompletely defined.

### 3.4. Metabolic and Mitochondrial Modulators

Mitochondrial dysfunction, altered redox homeostasis, and metabolic reprogramming influence both the establishment and persistence of cellular senescence. Dysfunctional mitochondria can generate excessive mitochondrial ROS, which in turn reinforces DNA damage, telomere instability, p38 MAPK activation, and p53–p21^CIP1^–pRB-mediated cell-cycle arrest [9,16,17]. Mitochondrial damage may therefore participate in a positive feedback loop in which metabolic dysfunction promotes senescence, while the senescent state further compromises mitochondrial homeostasis.

The mechanistic target of rapamycin (mTOR) and AMP-activated protein kinase (AMPK) are additional metabolic regulators implicated in senescence. mTOR integrates nutrient and growth-factor signals and can promote the translation of selected SASP components, whereas AMPK senses cellular energy status and may counterbalance metabolic stress and restrain senescence progression [44,45,46,47]. Through their effects on protein synthesis, autophagy, mitochondrial function, and cellular energy balance, these pathways may influence both the magnitude and persistence of the senescent phenotype. However, evidence supporting their roles in ocular-surface senescence is derived largely from general or non-ocular studies, and their cell-type-specific functions in ocular tissues remain to be directly established.

**Figure 1:**
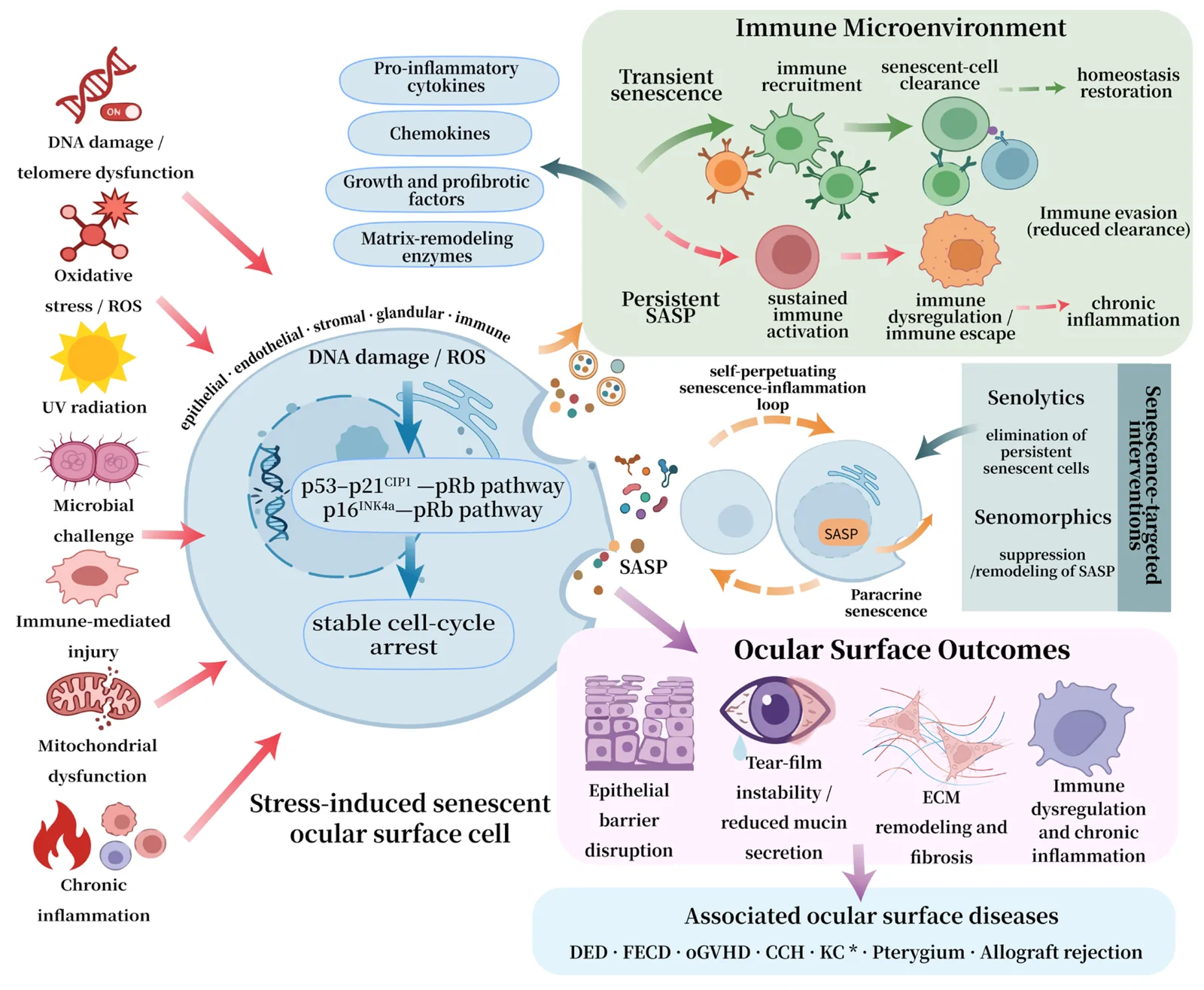
Integrated framework of stress-induced cellular senescence and SASP-mediated ocular surface pathology. Ocular surface stressors, including DNA damage, telomere dysfunction, oxidative stress, ultraviolet radiation, microbial challenge, immune-mediated injury, mitochondrial dysfunction, and chronic inflammation, promote cellular senescence through canonical cell-cycle-arrest pathways. Transient senescence may facilitate immune recruitment, senescent-cell clearance, and restoration of homeostasis, whereas persistent SASP signaling promotes paracrine senescence, sustained immune activation, immune evasion, epithelial barrier disruption, tear-film instability, extracellular-matrix remodeling, fibrosis, and chronic inflammation. Senolytics and senomorphics are shown as experimental strategies for reducing persistent senescent-cell burden or modifying SASP activity. Solid arrows indicate relationships supported by ocular experimental or clinical evidence, whereas dashed arrows indicate proposed mechanisms or relationships supported mainly by indirect evidence. * KC is included on the basis of indirect molecular associations; direct evidence of cellular senescence remains limited. Abbreviations: CCH, conjunctivochalasis; DED, dry eye disease; DNA, deoxyribonucleic acid; ECM, extracellular matrix; FECD, Fuchs endothelial corneal dystrophy; KC, keratoconus; oGVHD, ocular graft-versus-host disease; p16INK4a, cyclin-dependent kinase inhibitor p16INK4a; p21CIP1, cyclin-dependent kinase inhibitor p21CIP1; p53, tumor protein p53; pRB, retinoblastoma protein; ROS, reactive oxygen species; SASP, senescence-associated secretory phenotype.

Taken together, these findings support an integrated stress–senescence–SASP–immune remodeling axis that links ocular surface injury to tissue dysfunction and provides the biological rationale for senescence-targeted interventions, as summarized in Figure 1.

## 4. Cellular Senescence in Ocular Surface Diseases

### 4.1. Dry Eye Disease

DED is a multifactorial chronic OSD characterized by loss of tear-film homeostasis, ocular discomfort, visual disturbance, and ocular surface inflammation. Increasing evidence suggests that DED is not merely a tear-deficient disorder but a chronic inflammatory condition shaped by aging-related immune dysregulation, environmental stress, and ocular surface dysfunction [48,49].

Aging is an independent risk factor for DED [50,51]. Age-related changes in lacrimal gland function, meibomian gland structure, goblet-cell support, and ocular surface immunity create a susceptible background for disease [50,51]. Senescence-associated beta-galactosidase (SA-β-gal) positivity and activation of the p53–p21^CIP1^–pRB and p16^INK4a^–pRB pathways have been observed in aged lacrimal and meibomian glands, suggesting that cellular senescence may contribute to glandular remodeling and functional decline [24,52,53,54]. Desiccating and oxidative stress activate inflammatory mediators and matrix metalloproteinase-9 (MMP-9), disrupt epithelial barrier integrity, and amplify tear-film instability [20,55,56]. SIPS may therefore link environmental injury to chronic ocular surface inflammation, but whether it independently drives DED onset or progression remains uncertain. Future studies should identify the affected cell populations, determine whether senescent-cell accumulation precedes disease progression, and test whether targeted clearance restores ocular surface homeostasis.

### 4.2. Fuchs Endothelial Corneal Dystrophy

FECD is a genetically heterogeneous, progressive corneal endothelial disorder characterized by loss of corneal endothelial cells (CEnCs), Descemet membrane thickening, guttae formation, excessive ECM deposition, stromal edema, and progressive visual decline [26]. Reported senescence-associated features include oxidative stress, impaired DNA repair, increased p21^CIP1^ and p16^INK4a^ protein expression, SASP activation, and fibrotic matrix remodeling. Transcriptomic profiling of human FECD endothelium found 31 of 89 senescence-associated genes to be differentially expressed, including increased *CDKN2A* and NOX4 expression [23,57]. These findings suggest a potential link between oxidative stress and endothelial senescence.

In a Col8a2 mutant mouse model, Matthaei et al. observed increased *Cdkn1a* expression without a clear change in *Cdkn2a* expression, suggesting that a p53–p21^CIP1^–pRB-dominant stress response may predominate in this model [23,58]. Human late-onset FECD endothelium showed increased *CDKN2A* expression [57], but this finding alone does not establish irreversible senescence in a tissue with intrinsically limited proliferative capacity. In vitro, p16^INK4a^-positive senescent CEnCs promoted guttae-like structures and abnormal ECM deposition, whereas *CDKN2A* knockdown suppressed these changes [26]. Senescent CEnCs may also promote endothelial–mesenchymal transition and fibrosis through SASP-associated mediators, including IL-8 and IL-17 [11,59,60]. Ultraviolet A exposure can induce cell-cycle re-entry followed by G2/M arrest and generate senescence markers and fibrotic phenotypes, including alpha-smooth muscle actin expression [61]. Chronic stress may instead drive irreversible G0/G1 arrest through sustained ataxia telangiectasia mutated (ATM) activation; ATM inhibition restored repair capacity and reduced senescence burden in experimental models [62]. Overall, current evidence supports a role for cellular senescence in FECD-related endothelial dysfunction, guttae formation, and matrix remodeling, although its contribution to disease progression requires further clarification.

### 4.3. Ocular Graft-Versus-Host Disease

Ocular graft-versus-host disease (oGVHD) is a common and severe complication of allogeneic hematopoietic stem cell transplantation, with a reported prevalence of up to 60% among patients with graft-versus-host disease (GVHD). Aberrant activation of donor-derived immune cells leads to chronic inflammation and progressive fibrosis in multiple organs [63,64]. Cellular senescence has been implicated in chronic oGVHD. Ocular tissues from patients with oGVHD show DDR activation, increased p16^INK4a^ and p21^CIP1^ protein expression, mitochondrial dysfunction, and enhanced SASP activity [24,63]. IL-6 is among the SASP-associated mediators implicated in inflammatory and fibrotic remodeling in oGVHD [24,63].

In oGVHD-associated keratopathy, oxidative damage markers such as 8-hydroxy-2′-deoxyguanosine (8-OHdG) and 4-hydroxynonenal (4-HNE) are increased, whereas antioxidant defenses, including nuclear factor erythroid 2-related factor 2 (Nrf2) and catalase, are suppressed. p16^INK4a^ and p38 MAPK signaling are also activated, promoting NF-κB-mediated release of IL-6 and IL-8 [21]. Desiccating stress may reinforce this pathology by intensifying local alloimmune responses [63,65,66]. p16^INK4a^-positive senescent cells and mitochondrial dysfunction have been observed in lacrimal gland and conjunctival tissues [24,63]. SASP-associated mediators may contribute to fibroblast activation and fibrosis, and cellular senescence has also been implicated in chronic GVHD-associated meibomian gland dysfunction [53,63]. Thus, current evidence supports a role for senescence in chronic oGVHD, although some links remain supportive or indirect.

### 4.4. Corneal Allograft Rejection

Corneal transplantation remains the principal treatment for end-stage corneal blindness, but immune rejection is a leading cause of graft failure. Transplantation-related stress, endothelial vulnerability, inflammation, and oxidative injury may create a permissive environment for SIPS-like phenotypes [67,68]. In a murine penetrating keratoplasty model, graft endothelial cells showed DNA damage, ROS accumulation, activation of the p16^INK4a^–pRB pathway, SA-β-gal positivity, increased *CDKN2A* expression, and reduced proliferative capacity [67]. These findings support a senescence-associated endothelial response after transplantation, but they do not independently establish a stable senescent state in cells with intrinsically limited proliferative capacity. Stromal fibroblast senescence may limit proliferation early after injury but promote chronic inflammation and remodeling through the SASP at later stages [69].

As rejection progresses toward chronic graft failure, cytokines may cooperate with oxidative stress to promote senescence. In human corneal endothelial B4G12 cells, transforming growth factor beta 1 (TGF-β1) increased mitochondrial ROS and induced increased p16^INK4a^ and p21^CIP1^ protein expression, G1 arrest, and elevated SA-β-gal activity; N-acetyl-L-cysteine attenuated these changes [70]. Increased p16^INK4a^ and p27^KIP1^ protein expression has also been reported in selected renal allografts with rejection [71], and increased biliary p16^INK4a^, p21^CIP1^, and p53 protein expression has been observed in liver allograft rejection [72]. In experimental corneal transplantation, clearance of senescent cells restored angiotensin-converting enzyme 2 (ACE2), reduced SASP activity, and improved immune homeostasis [73]. Direct causal evidence in human corneal graft rejection nevertheless remains limited.

### 4.5. Other Ocular Surface Diseases

Pterygium is an ocular surface degenerative disorder associated with ultraviolet exposure and chronic oxidative stress. Increased IL-6, IL-8, matrix metalloproteinases, transforming growth factor beta, and vascular endothelial growth factor resemble a SASP-like profile and may contribute to epithelial hyperplasia, stromal remodeling, fibrovascular proliferation, and angiogenesis [74]. These mediators are not specific to senescence and may also reflect chronic inflammation or tissue remodeling. Current findings therefore provide indirect rather than definitive evidence. Confirmation requires cell-type-specific localization with complementary markers, evidence of stable cell-cycle arrest, and functional manipulation of senescent cells.

Keratoconus (KC) is increasingly recognized as a multifactorial corneal ectatic disorder involving oxidative stress, inflammation, ECM remodeling, and metabolic dysregulation [75]. Tear proteomic analysis by Kannan et al. identified reduced expression of antioxidant enzymes, including GSS and SOD1, together with alterations in inflammation-related and ECM-associated proteins. Pathway enrichment further implicated neutrophil degranulation, autophagy, and metabolic reprogramming [76]. However, canonical senescence markers and senescent-cell accumulation were not directly assessed. Thus, these findings suggest biological overlap between KC and senescence-related stress responses but do not establish cellular senescence or SASP signaling as causal mechanisms in KC.

CCH is common in older adults and is characterized by redundant conjunctival folds between the globe and lower eyelid. In conjunctival fibroblasts, increased intracellular ROS activates p38 MAPK and promotes SIPS through the p53–p21^CIP1^–pRB pathway [22]. These findings provide direct mechanistic evidence linking ROS–p38 MAPK signaling to fibroblast senescence and conjunctival remodeling. Larger clinical studies are needed to distinguish the contributions of senescence, age-related degeneration, and chronic inflammation.

Representative studies linking cellular senescence and stress-related mechanisms to ocular surface diseases are summarized in Table 1.

## 5. Role of Senescence in Ocular Surface Homeostasis

### 5.1. Dual Roles of Senescence in Ocular Surface Physiology and Pathology

Under physiological conditions, ocular surface homeostasis depends on the coordinated regulation of barrier, secretory, immune, neural, stromal, and local microecological functions. Corneal and conjunctival epithelial cells maintain surface integrity; goblet cells stabilize the tear film through mucin secretion; corneal endothelial cells (CEnCs) preserve stromal deturgescence; sensory nerves regulate blinking and lacrimal secretion; and resident immune cells, including dendritic cells, macrophages, γδ T cells, natural killer (NK)/natural killer T (NKT) cells, CD8^+^ T cells, and B cells, together with regulatory cytokines such as TGF-β and IL-10, maintain immune surveillance without excessive inflammation [48,49,79]. This integrated view provides the basis for understanding why senescence can be either adaptive or harmful depending on its duration and clearance.

Cellular senescence is not necessarily pathological. A transient response may limit aberrant proliferation of damaged cells and coordinate tissue repair. Senescent fibroblasts have been observed during mouse corneal wound healing [69], although proposed benefits of senescence-associated platelet-derived growth factor AA secretion derive from non-ocular wound-healing models and require direct validation in the cornea [5,80]. Effective immune clearance prevents excessive senescent-cell accumulation and allows repair-associated SASP signals to resolve [48].

When stress persists or immune clearance fails, the reparative response may become maladaptive. Persistent SASP signaling can impair epithelial renewal, destabilize the tear film, activate fibroblasts, disrupt immune tolerance, and reinforce paracrine senescence [11,37,39,81,82]. The biological effect of senescence therefore depends on its duration, cellular context, and efficiency of immune clearance.

### 5.2. Senescence-Driven Cell-Type-Specific Pathologies

Cellular senescence exerts cell-type-specific effects on ocular surface homeostasis by altering the functional properties of epithelial, endothelial, stromal, secretory, and immune cells. In corneal epithelial cells, stress-induced senescence reduces proliferative reserve, delays re-epithelialization, weakens barrier integrity, and impairs epithelial stem cell function and cell migration [77,83]. SASP-mediated inflammatory signaling further disrupts epithelial homeostasis, and TGF-β can induce corneal epithelial senescence and canonical SASP production; inhibition of this pathway partially reverses the associated phenotype [78]. CEnCs are particularly susceptible to senescence because of their limited regenerative capacity in vivo. Senescence reduces endothelial cell density and pump reserve, increases cellular pleomorphism and polymegathism, and compromises stromal deturgescence, thereby predisposing the cornea to edema and endothelial decompensation [23]. Upregulation of p21^CIP1^ further supports the involvement of premature senescence in endothelial functional decline, although its causal contribution remains to be fully validated.

In corneal fibroblasts, persistent senescence can promote inflammatory and matrix-remodeling phenotypes, although senescent fibroblasts may also limit fibrosis in specific wound-healing contexts [24,25,69]. Direct evidence for senescence in conjunctival goblet cells remains limited; chronic inflammation and oxidative stress may impair mucin secretion and tear-film stability. Immune-cell senescence may further reduce immune regulation and senescent-cell clearance. Senescent T cells can exhibit functional exhaustion and impaired immunoregulatory activity, potentially weakening local immune tolerance [10,38,48].

Under pathological stress, cellular senescence is not merely a passive marker of tissue injury. Rather, it alters the core functions of ocular surface cells, including epithelial renewal, barrier maintenance, endothelial pump activity, stromal remodeling, mucin secretion, and immune surveillance. This stress–senescence–functional decline axis provides a useful framework for understanding how ocular surface homeostasis becomes progressively destabilized [11,13,14].

## 6. Therapeutic Strategies Targeting Senescence

### 6.1. Senolytics

Senolytics are agents designed to selectively eliminate senescent cells by inducing apoptosis, thereby reducing senescent-cell burden and attenuating detrimental SASP signaling [84,85]. Representative senolytics include B-cell lymphoma 2 (BCL-2) family inhibitors such as navitoclax (ABT-263), tyrosine kinase inhibitors such as dasatinib, natural compounds such as quercetin and fisetin, and newer investigational agents such as UBX-1325 [84,86]. However, most evidence for these compounds derives from systemic aging models, non-ocular diseases, or ocular diseases outside the ocular surface, rather than from OSD models [84,85,86].

Direct preclinical evidence for senolytics in ocular surface and adnexal models remains limited but is emerging. In the p16-3MR mouse model, ganciclovir-mediated ablation of p16^INK4a^-positive cells reduced senescence- and SASP-related gene expression, corneal opacity, and angiogenesis after lacrimal gland excision [13]. Because ablation depends on the engineered p16-3MR/HSV-TK system, this result is proof of concept rather than evidence for pharmacological treatment of DED. In chronic oGVHD models, systemic navitoclax (ABT-263) reduced senescence-associated markers, inflammation, and fibrosis in lacrimal and meibomian glands [24,53]. These studies support further evaluation of senescent-cell clearance, but efficacy and safety in human disease remain unestablished [85,87,88].

Senolytics may also be relevant to corneal transplantation. In a murine model, systemic ABT-263 reduced alloimmune responses, and its protective effect was closely linked to ACE2 signaling [73]. Whether these findings translate to human transplantation is unknown. Translation also requires caution because transient senescence may support wound repair, ocular cell types may differ in senolytic sensitivity, and systemic BCL-2 family inhibitors can cause hematologic toxicity [84,85]. Future studies should define timing, dose, delivery route, cell-type specificity, and long-term ocular safety. In another ocular tissue, BST2-targeted senotherapy restored visual function in a preclinical retinal model [89], but its relevance to OSDs is unknown.

### 6.2. Senomorphics

Unlike senolytics, which eliminate senescent cells, senomorphics suppress or reshape the SASP. This approach may be relevant to OSDs because epithelial, glandular, endothelial, and stromal integrity is essential for tear-film stability, barrier function, corneal transparency, and immune homeostasis.

Several pathways are candidates for SASP modulation. mTOR regulates SASP production [44], but the value of mTOR-directed therapy in OSDs has not been established [84,90]. Janus kinase (JAK)/signal transducer and activator of transcription (STAT) inhibition have shown senomorphic effects in non-ocular models, including suppression of JAK2/STAT3-associated senescence [91]. In a murine chronic GVHD model, an anti-IL-6 receptor antibody reduced lacrimal gland fibrosis [24], supporting the potential of targeting this SASP-associated cytokine pathway. These findings do not distinguish selective senomorphic effects from broader anti-inflammatory actions [63].

NF-κB integrates signals from the DDR, cGAS–STING, oxidative stress, and inflammatory pathways to drive SASP gene expression. Bromodomain-containing protein 4 (BRD4) and other epigenetic regulators can cooperate with NF-κB to sustain this transcriptional program [92,93]. Their therapeutic relevance in OSDs remains unproven. Delivery is an additional constraint: systemic treatment can increase off-target toxicity, whereas topical delivery is limited by tear turnover, blinking, nasolacrimal drainage, tissue barriers, and short precorneal residence time [94]. Periocular or local injection may increase exposure but introduces risks of injury, inflammation, clearance, and tissue toxicity [95]. Future ocular senotherapy must therefore optimize delivery, retention, tissue specificity, and safety without disrupting physiological repair [13,24,53,73,84].

## 7. Conclusions and Perspectives

Current evidence supports cellular senescence as a context-dependent link between ocular surface stress, inflammation, cellular dysfunction, and tissue remodeling. Direct mechanistic contributions have been demonstrated in selected experimental models, whereas evidence in several human OSDs remains associative. Senolytics and senomorphics require further validation of ocular safety, cell-type specificity, timing, and delivery. Future studies should define disease- and cell-specific markers, use cell-resolved profiling to identify senescent populations, and test whether selective depletion or functional modulation improves disease outcomes.

## Figures and Tables

**Table 1:** Representative studies linking cellular senescence and stress-related mechanisms to ocular surface diseases.

Disease	Study and Design	Overall Level of Evidence	Senescence Markers or Criteria Examined	Model/Sample	Evidence-Supported Finding	Ref.
	López-Miguel et al. (2014); prospective study	Indirect (primary study)	Not directly assessed; corneal staining, tear-film breakup time, and tear MMP-9 were evaluated following desiccating stress.	Nineteen patients with dry eye disease and 20 asymptomatic controls; 2 h at 5% relative humidity	Acute desiccating stress increased corneal staining and tear matrix metalloproteinase-9 and reduced tear-film breakup time in both groups. Cellular senescence was not directly assessed.	[56]
Dry eye disease	Galletti and de Paiva (2021); narrative review	Indirect (review)	Not applicable; narrative review without original experimental assessment of senescence markers.	Synthesis of experimental and clinical evidence on aging of the ocular surface immune system	Aging alters ocular surface immune regulation and is associated with a higher incidence of ocular surface disease. This article is a review, not a primary animal experiment.	[48]
	Kitazawa et al. (2022); systematic review and meta-analysis	Indirect (systematic review and meta-analysis)	Not directly assessed; age-related dry-eye prevalence and clinical ocular-surface changes were evaluated.	Forty-four studies of age-related ocular changes and 14 epidemiological studies; eight studies included in a random-effects meta-analysis	In adults aged ≥60 years, pooled dry eye disease prevalence was 9.2%, and the odds ratio versus younger adults was 1.313. Aging was also associated with tear-film disruption, inflammation, and senescence-related tissue changes.	[50]
	Matthaei et al. (2012); translational study	Supportive	SA-β-gal activity; p53 and p21^CIP1^ expression; senescence-associated transcripts, including *Cdkn1a*, Serpine1, Tagln, Fn1, and Clu.	Col8a2Q455K/Q455K mice and human Fuchs endothelial corneal dystrophy specimens	Mutant mouse endothelium showed increased p53–p21^CIP1^–pRB signaling and senescence markers; p21^CIP1^ was also increased in human disease tissue, supporting premature endothelial senescence.	[23]
	Matthaei et al. (2013); transcriptomic study	Indirect (primary study)	No direct senescence assay; whole-genome stress-response profiling, including COX-2 and JUN expression.	Col8a2Q455K/Q455K mouse endothelium with validation in human specimens	The model showed broad stress-response changes and increased COX-2 and JUN expression; COX-2 was also increased in human tissue. The study did not report ATM pathway upregulation.	[58]
Fuchs endothelial corneal dystrophy (FECD)	Matthaei et al. (2014); human case–control tissue study	Supportive	Senescence-associated transcript profiling, including *CDKN2A*/p16^INK4a^ and NOX4; no direct assessment of stable cell-cycle arrest.	Endothelium from 21 affected patients and 12 normal autopsy eyes	NOX4 and the *CDKN2A* pathway were upregulated, linking oxidative stress and cellular senescence to diseased endothelium.	[57]
	White et al. (2021); in vitro and in vivo mechanistic study	Direct	Cell-cycle re-entry followed by G2/M arrest; DNA-damage and senescence-associated chromatin markers; response to ATR inhibition and senolytic treatment.	Ultraviolet A-induced human corneal endothelial cell and animal models	Ultraviolet A induced G2/M arrest, senescence, endothelial-to-mesenchymal transition, and fibrosis. ATR inhibition or senolytic treatment attenuated the profibrotic phenotype.	[61]
	Ashraf et al. (2024); mechanistic stress-model study	Direct	Ki67-associated cell-cycle re-entry; G2/M versus G0/G1 arrest; pATM, pH2AX, and H3K9me3; response to ATM inhibition.	Normal and NQO1-deficient human corneal endothelial cells and Atm-null mice exposed to menadione, ultraviolet A, or 4-hydroxyestradiol	Acute ATM activation promoted G2/M repair and cytoprotective arrest, whereas sustained ATM activation under chronic stress promoted G0/G1 arrest and cytotoxic senescence; ATM inhibition restored repair in the chronic model.	[62]
	Parekh et al. (2025); long-term in vitro model	Direct	*CDKN2A*/p16^INK4a^ expression, SA-β-gal activity, persistent G0/G1 arrest after long-term recovery, and response to p16^INK4a^ knockdown.	Immortalized human corneal endothelial cells repeatedly exposed to ultraviolet A and 4-hydroxyestradiol, followed by approximately 60 days of recovery	Persistent p16^INK4a^-positive G0/G1 senescence promoted pathological extracellular-matrix deposition and guttae-like formation.	[26]
	Parekh et al. (2026); chronic in vitro stress model	Direct	p16^INK4a^–pRB activation, p21^CIP1^ expression, SA-β-gal activity, G0/G1 arrest, SASP factors including IL-8 and IL-17, and responses to cytokine blockade and dasatinib plus quercetin.	Human corneal endothelial cells exposed to ultraviolet A and 4-hydroxyestradiol	p16^INK4a^–pRB-mediated G0/G1 arrest induced a senescence-associated secretory phenotype and fibrosis. Cytokine blockade and dasatinib plus quercetin reduced pathological changes.	[60]
	Yamane et al. (2020); mouse and human translational study	Direct	p16^INK4a^-positive senescent cells, mitochondrial alterations, SASP factors including IL-6 and CXCL9, and response to ABT-263-mediated senolysis.	Sclerodermatous chronic graft-versus-host disease mouse model and human lacrimal gland specimens	Senescent cells and interleukin-6/CXCL9-associated secretory factors accumulated in lacrimal glands; ABT-263-mediated senolysis improved gland pathology. STAT3 was not established in the abstract as the causal pathway.	[24]
Ocular graft-versus-host disease (oGVHD)	Gehlsen et al. (2022); retrospective cohort study	Indirect (primary study)	Not directly assessed; environmental humidity and the occurrence, severity, and onset of ocular GVHD were evaluated.	Four hundred forty-four allogeneic hematopoietic stem-cell transplant recipients; ward humidity correlated with disease occurrence, severity, and onset	Low relative humidity was an independent risk factor: 73% developed ocular graft-versus-host disease after transplantation under desiccating conditions versus 30% under high humidity. Cellular senescence was not directly assessed.	[65]
	Sato et al. (2024); allogeneic bone-marrow-transplant mouse study	Direct	*CDKN2A*/p16^INK4a^ and other senescence-associated markers, SASP-factor expression, and response to ABT-263-mediated senolysis.	Chronic graft-versus-host disease mice treated with ABT-263	Meibomian-gland atrophy, inflammation, fibrosis, senescence markers, and secretory factors increased in disease; ABT-263 preserved gland area and reduced pathology.	[53]
	He et al. (2024); single-cell transcriptomic study	Indirect (primary study)	Not directly assessed; single-cell transcriptional changes, immune-cell composition, and inflammatory pathways were examined.	Lacrimal glands from a mouse graft-versus-host disease model; single-cell RNA sequencing, bioinformatics, and immunofluorescence validation	Acinar, myoepithelial, and endothelial populations decreased, whereas T cells and macrophages expanded, with a T-helper-2-linked inflammatory pattern. Cellular senescence was not directly tested.	[64]
	Zhao et al. (2016); mouse, human-tissue, and in vitro study	Supportive	SA-β-gal activity, p16^INK4a^ expression, oxidative-stress and antioxidant responses, reduced proliferative capacity, and ROS–ASK1–p38 MAPK signaling.	Normal-risk orthotopic mouse transplantation, human graft endothelium, and hydrogen-peroxide-treated human corneal endothelial cells	Allograft endothelium showed oxidative imbalance, p16^INK4a^ upregulation, and premature senescence; in cultured cells, reactive oxygen species induced p16^INK4a^ through the ASK1–p38 MAPK pathway.	[67]
Post-penetrating keratoplasty	Li et al. (2018); human tissue and in vitro mechanistic study	Direct	p16^INK4a^ and p21^CIP1^ expression; G1-phase arrest; SA-β-gal activity; SASP-factor expression; mitochondrial ROS accumulation; and response to NAC treatment	Human corneal buttons with chronic graft failure; a murine chronic corneal allograft-failure model; and human corneal endothelial B4G12 cells treated with TGF-β1, with or without NAC	Senescence-associated changes were detected in chronic corneal allograft failure. In B4G12 cells, TGF-β1 increased mitochondrial ROS and induced a senescence-associated phenotype, whereas NAC attenuated these changes.	[70]
	Chi et al. (2025); mechanistic mouse transplantation study	Direct	SA-β-gal activity and p16^INK4a^ expression; p16^INK4a^ knockout, ABT-263-mediated senolysis, adoptive transfer, and ACE2 loss-of-function experiments.	Murine corneal transplantation with p16^INK4a^ knockout, adoptive transfer, ABT-263 treatment, and ACE2 loss-of-function	Transplantation stress-induced senescence aggravated rejection; ABT-263 suppressed alloresponses, and its anti-rejection effect depended closely on angiotensin-converting enzyme 2.	[73]
Corneal and conjunctival epithelial aging	Kitazawa et al. (2023); in vitro and human-tissue study	Direct	Flattened cell morphology, SA-β-gal activity, reduced EdU incorporation, p16^INK4a^ and p21^CIP1^ expression, SASP-associated transcription, and tissue p16^INK4a^/Ki67 staining.	X-irradiated human corneal and conjunctival epithelial cells and pathological conjunctival specimens	Both epithelial cell types acquired canonical senescence features but distinct expression profiles; senescent conjunctival epithelial cells were detected in severe limbal stem-cell-deficiency tissues.	[77]
	Li et al. (2016); human tissue and in vitro mechanistic study	Direct	G1 arrest, SA-β-gal activity, p16^INK4a^ and p21^CIP1^ expression, SASP factors including IL-6, IL-8, and TNF-α, and reversal by TGF-β or NF-κB inhibition.	Elderly donor corneal epithelium and human corneal epithelial cell models; transforming growth factor-β and NF-κB perturbation	Transforming growth factor-β promoted epithelial senescence and inflammation through NF-κB activation and accelerated IκBα mRNA degradation; NF-κB blockade attenuated the phenotype.	[78]
Corneal wound healing and fibrosis	Wang et al. (2019); mouse and in vitro study	Direct	SA-β-gal activity, p16^INK4a^ expression, reduced mitogenic responses, and senescence-associated matrix-remodeling genes.	Mouse epithelial-debridement, scarring, alkali-burn, and keratoplasty models plus cultured fibroblasts	Senescent stromal fibroblasts displayed a nonfibrogenic phenotype, with increased MMP-1/3/13 and reduced fibronectin and collagen I, and may help limit corneal fibrosis.	[69]
Keratoconus	Kannan et al. (2025); human tear-proteomics study	Indirect (primary study)	Not directly assessed; tear-proteomic changes related to oxidative defense, inflammation, extracellular-matrix remodeling, and metabolism were examined.	Tears from 40 controls and 107 eyes from 69 patients	Thirty-two tear proteins were dysregulated, implicating inflammation, extracellular-matrix organization, oxidative defense, and metabolism. The study proposed progression markers but did not directly evaluate cellular senescence.	[76]
Conjunctivochalasis	Xiang et al. (2019); patient-tissue and in vitro study	Direct	SA-β-gal activity, intracellular ROS, p38 activation, p53 and p21^CIP1^ expression, and response to p38 inhibition.	Conjunctiva and fibroblasts from 13 patients and 12 age-matched controls	Patient tissue showed more senescent cells and higher p38, p53, and p21^CIP1^ expression; p38 blockade reduced reactive oxygen species and senescence, supporting p38–p53–p21^CIP1^–pRB signaling.	[22]

Abbreviations: 4-OHE2, 4-hydroxyestradiol; ACE2, angiotensin-converting enzyme 2; ATM, ataxia telangiectasia mutated; ATR, ataxia telangiectasia and Rad3-related protein; COX-2, cyclooxygenase-2; CXCL9, interferon-γ-inducible chemokine 9; ECM, extracellular matrix; FECD, Fuchs endothelial corneal dystrophy; IL, interleukin; IκBα, inhibitor of nuclear factor kappa B alpha; iTRAQ, isobaric tags for relative and absolute quantitation; LC–MS/MS, liquid chromatography–tandem mass spectrometry; MMP, matrix metalloproteinase; NF-κB, nuclear factor kappa B; NQO1, NAD(P)H quinone dehydrogenase 1; oGVHD, ocular graft-versus-host disease; p38 MAPK, p38 mitogen-activated protein kinase; PCR, polymerase chain reaction; pRB, retinoblastoma protein; RNA, ribonucleic acid; SA-β-gal, senescence-associated β-galactosidase; TGF-β, transforming growth factor beta; UVA, ultraviolet A.

## Data Availability

No new data were created or analyzed in this study. Data sharing is not applicable to this article.
